# Development of Hydrophilic PVDF Membrane Using Vapour Induced Phase Separation Method for Produced Water Treatment

**DOI:** 10.3390/membranes10060121

**Published:** 2020-06-16

**Authors:** Normi Izati Mat Nawi, Ho Min Chean, Norazanita Shamsuddin, Muhammad Roil Bilad, Thanitporn Narkkun, Kajornsak Faungnawakij, Asim Laeeq Khan

**Affiliations:** 1 Department of Chemical Engineering, Universiti Teknologi PETRONAS, Bandar Seri Iskandar, Perak 32610, Malaysia; normi_16000457@utp.edu.my (N.I.M.N.); ho.min_22670@utp.edu.my (H.M.C.); 2 Faculty of Integrated Technologies, Universiti Brunei Darussalam, Jalan Tungku Link, Gadong BE 1410, Brunei; norazanita.shamsudin@ubd.edu.bn; 3 National Nanotechnology Center (NANOTEC), National Science and Technology Development Agency (NSTDA), 111 Thailand Science Park, Pathum Thani 12120, Thailand; thanitporn.nar@ncr.nstda.or.th (T.N.); kajornsak@nanotec.or.th (K.F.); 4 Department of Chemical Engineering, COMSATS University Islamabad, Lahore Campus, Islamabad 45550, Pakistan; alaeeqkhan@cuilahore.edu.pk

**Keywords:** vapor induced phase separation, membrane fabrication, hydrophilic, oily wastewater, antifouling

## Abstract

During the production of oil and gas, a large amount of oily wastewater is generated, which would pollute the environment if discharged without proper treatment. As one of the most promising treatment options, membrane material used for oily wastewater treatment should possess desirable properties of high hydraulic performance combined with high membrane fouling resistance. This project employs the vapor induced phase separation (VIPS) technique to develop a hydrophilic polyvinylidene fluoride (PVDF) membrane with polyethylene glycol (PEG) as an additive for produced water treatment. Results show that thanks to its slow nonsolvent intake, the VIPS method hinders additive leaching during the cast film immersion. The results also reveal that the exposure of the film to the open air before immersion greatly influences the structure of the developed membranes. By extending the exposure time from 0 to 30 min, the membrane morphology change from typical asymmetric with large macrovoids to the macrovoid-free porous symmetric membrane with a granular structure, which corresponds to 35% increment of steady-state permeability to 189 L·m^−2^h^−1^bar^−1^, while maintaining >90% of oil rejection. It was also found that more PEG content resides in the membrane matrix when the exposure time is extended, contributes to the elevation of surface hydrophilicity, which improves the membrane antifouling properties. Overall results demonstrate the potential of VIPS method for the fabrication of hydrophilic PVDF membrane by helping to preserve hydrophilic additive in the membrane matrices.

## 1. Introduction

Oil and gas are some of the most important industries to supply energy demands. However, they are accountable for water contamination during the refinery and/or production operation. One of the largest byproducts of oil and gas production and processing is produced water (PW). PW that contains emulsified oil/water and when highly contaminated can tremendously pollute the environment. The separation of these emulsified oil/water mixtures is difficult, and it is typically done by using a combination of a few conventional processes such as settlement and centrifugation as well as dissolved air flotation. 

Membrane technologies have achieved a remarkable improvement over the last few years in terms of salt rejection, water permeability, and quality of treated permeate. It is known to offer excellent oil separation efficiency, especially dealing with stable emulsified oil in the PW treatment. For instance, Akdemir and Ozer [1] employed a flat sheet PVDF membrane for separation of emulsified oil that resulted in an oil separation efficiency of up to 98.53%. However, despite the great advantages offered, the practical application of membrane in industries is still limited by the inevitable membrane fouling issue.

To properly address the membrane fouling issue, researchers have been focusing on developing effective materials that can minimize fouling phenomena while offering high process stability with high oil rejection. Among all available polymeric based membrane, PVDF membrane has emerged out as the most widely used material due to its outstanding properties [2], but the plain PVDF suffer from membrane fouling due to its hydrophobic inherent property. Studies show that hydrophilic property can suppress membrane fouling propensity by limiting the interaction between oil (in the PW) with the membrane surface [3]. Hence, various techniques have been reported to hydrophilize the membrane surface including (i) blending of polymers with different properties with respect to their hydrophilicity/hydrophobicity, (ii) grafting hydrophilic branches on hydrophobic polymer backbones, and (iii) incorporation of a hydrophilic film on hydrophobic materials [4]. 

Fabrication of typical PVDF membrane has often been done by the nonsolvent induced phase separation (NIPS) with the addition of hydrophilic polymer additives. NIPS involves the conversion of the polymer solution to a two-phase system of solid polymer-rich phase and liquid polymer-poor phase to form membrane structure and membrane pores, respectively [5], by immediately immersing the casted dope solution into the nonsolvent. According to Reuvers et al. [6], the membrane formed through NIPS involves either instantaneous or delayed demixing, in which the former leads to the formation of highly porous substructure with microvoids, while the latter produces membranes with a porous macrovoid-free (often closed cell) substructure. 

Unlike NIPS, the vapor-induced phase separation (VIPS) technique is a relatively slow process that allows a more uniform diffusion of vapor into the polymer [7], which offers better control in the phase inversion process. In the VIPS, the cast film is exposed to an environment (typically open-air) with controlled humidity. The penetration of nonsolvent into the film causes the polymer to precipitate, which results in the formation of a thin skin layer of a symmetric porous membrane. Yushkin et al. [8] developed polyacrylonitrile (PAN) membrane using VIPS technique and found that all membranes exposed in water vapor possess a sponge-like structure to give the permeance of 74–405 kg·m^−2^h^−1^bar^−1^ different from a finger-like structure obtained by NIPS method. AlMarzooqi et al. [9] applied VIPS to fabricate PVDF membrane for membrane distillation and found that the extension of exposure time before immersion alters the film structure from amorphous, flat and smooth to rough and lumpy morphology due to solid-liquid phase separation (crystallization) of semi-crystalline PVDF during exposure under humid atmosphere.

One of the most common methods to produce hydrophilic property from hydrophobic polymer-based membranes (such as PVDF) is by incorporating hydrophilic additive in the dope solution. It is one of the simplest and straight forward ways to enhance membrane hydrophilicity [10,11]. Although the presence of addition in the dope solution complicates the ternary system, it has been widely proved effective to promote pore formation as well as to improve the membrane hydrophilicity [12]. Apparently, the most common organic additives that used to increase the membrane hydrophilicity are polyvinylpyrrolidone (PVP) and polyethylene glycol (PEG) [13]. The addition of the high molecular weight of PVP resulted in the formation of a thicker top layer with a dense sub membrane layer and suppressed the formation of macrovoids [14]. On the other hand, the increasing amount of PEG content not only enhanced hydrophilicity and water permeability, but also led to the formation of a more porous structure [13]. The PEG as additives in the PVDF-based membrane acts more likely as a pores promoter rather than residing as a hydrophilic protective layer on the membrane surface towards oily wastewater. When loaded in the dope solution, like PVP [15], PEG leaches out from the membrane matrix when casted polymer membrane was immersed in the coagulation bath [16,17,18,19]. The leaching of PEG from the membrane matrix assists in the formation of a highly porous membrane. In other words, PEG additive predominantly enhances the resulting membrane properties by improving the structural parameter, and less so on its impact on increasing surface tension due to the leaching phenomenon.

To minimize the PEG additive leaching, this study employs a simple and yet effective VIPS method to develop hydrophilic PVDF-based membranes with desirable properties for PW treatment. The slower nonsolvent intake rate in VIPS would favor the solid-liquid demixing [20], the preformation of the immobile top layer during exposure to humid air would act as the gate to prevent excessive leaching of PEG to the nonsolvent bath during the immersion step. We hypothesize that extending the exposure time of cast film under a humid atmosphere before immersion in coagulation would facilitate the formation of the immobile membrane layer on top of the cast film that hinders the mobility of PEG molecules to the nonsolvent which then hold the hydrophilic additive from leaching [7]. Hence, an investigation of the PVDF formation membrane with the presence of PEG additives via VIPS was investigated in this study. Detailed characterizations to prove the hypothesis were also conducted in terms of structural parameters and surface chemistry. Finally, membrane performance for PW treatment, as well as their antifouling properties, were also investigated. 

## 2. Materials and Methods 

### 2.1. Membrane Preparation

PVDF with a molecular weight of 534 kDa by GPC (Sigma–Aldrich, St. Louis, MO, USA) at 12 wt% was used as a polymer. Lithium chloride (LiCl, ACROS Organics, Belgium) and PEG (Sigma–Aldrich, St. Louis, MO, USA) with MW of 20 kDa were used as additives with compositions of 0.1 wt% and 3 wt% respectively [21]. Dimethylacetamide (DMAC, Sigma–Aldrich, St. Louis, MO, USA) of 81.9 wt% was used as the solvent. All the materials were homogeneously stirred and mixed for at least 24 h at a dissolution temperature of 60 °C. The polymer solution was sonicated for 24 h to remove the air bubbles to avoid membrane defects. 

**Figure 1:**
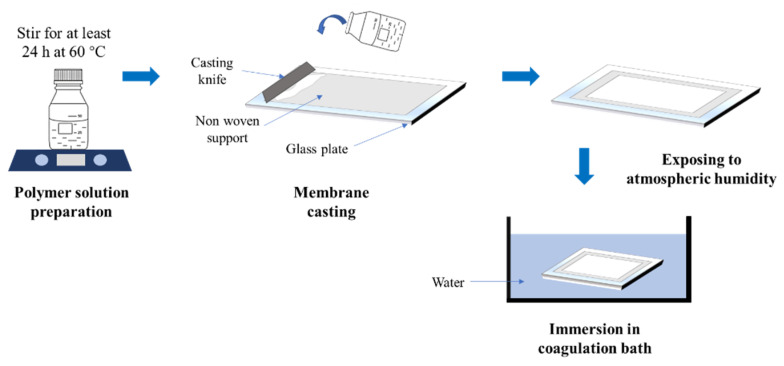
Simplified process flow of membrane preparation using VIPS method.

Figure 1 shows the illustration of the process flow diagram for membrane preparation using the VIPS method. The polymer solution was cast on nonwoven support (Novatexx 24413, Fredenberg-filter, Weinheim, Germany) to avoid shrinkage [22] using a casting knife with a wet thickness of 220 μm. The cast film was then exposed to the controlled room humidity of about 70% RH for the varied duration of 0, 5, 15, and 30 min before being immersed in a coagulation bath containing water. The resulting membranes were labeled as PVDF/PEG-0, PVDF/PEG-5, PVDF/PEG-15, and PVDF/PEG-30, respectively, according to the duration of the exposure. The cast film was later immersed in the coagulation bath containing water as nonsolvent for at least 24 h before used to ensure complete removal of solvent from the membrane matric and were kept wet until used for filtration. An additional plain PVDF membrane (of 12 wt%) without additive was also fabricated using a normal NIPS method as a benchmark for this study. 

### 2.2. Feed Preparation

Synthetic PW was prepared by mixing the deionized water and crude oil sample (obtained from one of the petrochemical industries in Malaysia) with the addition of a synthesis grade of sodium dodecyl sulfate (SDS 98%, Sigma–Aldrich, St. Louis, MO, USA). The small amount of SDS, with a ratio of 1:9 (w/w) of SDS to crude oil, acted as a surfactant to solubilize the oil in the water. A 1000 ppm (1 g/L) of oil/water emulsion was prepared by using a mechanical stirrer, stirred at a rate of 500 rpm for at least 24 h or until a homogeneously milky yellowish color suspension was obtained. In order to prevent the floatation/separation of the oil droplets (which might occur during storage period), the synthetic PW always prepared one day earlier prior to the filtration to ensure the consistency of the feed solution. 

### 2.3. Membrane Characterization

The morphological properties of the resulting membranes were observed using field emission scanning electron (FESEM, ZEISS EVO^®^ LS 15, Oberkochen, Germany). Prior to testing, the cross-section membrane samples were immersed in liquid nitrogen before cutting with a blade in order to minimize the shear effect due to membrane cutting. The pore size distribution of the resulting membranes was measured using a capillary flow porometer by Porolux 1000 (Porolux 1000, Berlin, Germany). A goniometer (Ramé-Hart 260, Succasunna, NJ, USA) was used to investigate the hydrophilicity of the membrane’s surface. The surface chemical compositions of the resulting membranes were studied using an X-ray photoelectron spectrometer (XPS, K-Alpha^TM^, Thermo Scientific, Waltham, MA, USA) while Fourier transform infrared spectrometer (FTIR) (PerkinELmer, Inc., Waltham, MA, USA) with the spectra range of 400 to 4000 cm^−1^ was used to analyze chemical bonds of the samples.

In addition, the turbidity of the feed and permeate samples were measured using a turbidity meter (HI-98703, Hanna Instruments, Woonsocket, RI, USA). A UV-VIS spectrometer (Shimadzu UV-2600, Kyoto, Japan) was used to determine the concentration of oil content of the samples at a wavelength of 227 nm. 

### 2.4. Filtration Configuration

**Figure 2:**
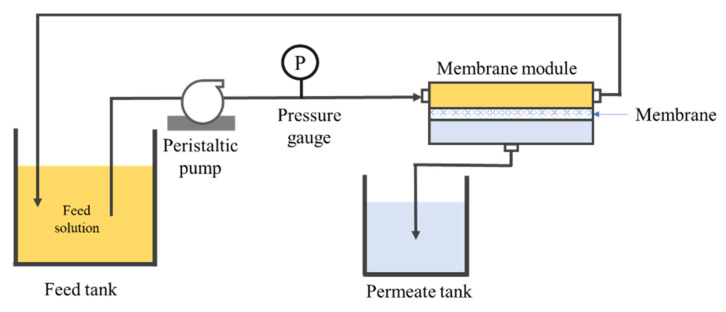
Schematic diagram of the crossflow filtration setup.

Figure 2 illustrates the schematic diagram of the crossflow filtration setup used for this study to investigate the hydraulic performance of the resulting membranes in treating synthetic PW. The filtration system was equipped with a peristaltic pump to circulate the feed through the system while maintaining the transmembrane pressure (Δ*P*) at 0.2 bar for each experiment. We deliberately applied a low-pressure for filtration test in this work since such operation is seen as a promising alternative mode to handle long-term membrane fouling. The low-pressure system allows operation under sustainable flux and less susceptibility from membrane fouling, as well as reduces the pumping energy consumption, as reported elsewhere [23,24]. A membrane sheet with an effective area of 34 cm^2^ was installed in the membrane cell. The membrane was first tested using distilled water for 1.5 h to evaluate its clean water permeability (CWP). 

For each membrane sample, the experiment using synthetic PW as feed was conducted for 1.5 h, and the permeate was collected for every 30 min. The collected permeate was returned into the feed tank to maintain the feed concentration. The membrane permeability and rejection performance were calculated using Equations (1) and (2), respectively.

(1) $$L=\frac{\Delta V}{A\;\Delta t\;\Delta P}$$

(2) $$R=\left(1-\frac{C_{p}}{C_{f}}\right)\times 100$$

where *L* represents the permeability (L m^−2^h^−1^bar^−1^), Δ*V* is the volume of permeate collected (L), *A* is the effective membrane area (m^2^), Δ*t* is the filtration time (h), Δ*P* is the transmembrane pressure (bar), *R* is oil/turbidity rejection (%), *C_p_* is the concentration of permeate (g/L), and *C_f_* is the concentration of feed solution (g/L). 

### 2.5. Membrane Fouling Resistance Test 

The permeability recovery ratio was determined by performing membrane washing with distilled water for 5 min after every 30 min of PW filtration. The combination of 30 min filtration followed by 5 min of membrane washing is considered as one complete cycle, and it was repeated for five cycles for each membrane samples. After compaction, PW filtration was performed for 90 min before the cleaning cycle was implemented as conditioning before the full cycle of filtration, and water flushing was implemented. Reversible and irreversible fouling was evaluated based on the filtration permeance obtained from each filtration cycle. Equations (3)–(5) were used to calculate the fouling resistance performance.

(3) $$IF=\frac{L_{0\left(n-1\right)}-L_{0\left(n\right)}}{L_{0\left(n-1\right)}}\times 100$$

(4) $$TF=\frac{L_{0\left(n\right)}-L_{\left(n\right)}}{L_{0\left(n\right)}}\times 100$$

(5) RF=TF−IF

where *IF* is irreversible fouling (%), *TF* is total fouling (%), *RF* if reversible fouling (%), *L*_0_ is clean water permeability, *L* is PW water permeability, and *n* is the number of filtration cycle (*n*^th^).

## 3. Results and Discussion

### 3.1. Membrane Characterizations

#### 3.1.1. Membrane Morphology

**Figure 3:**
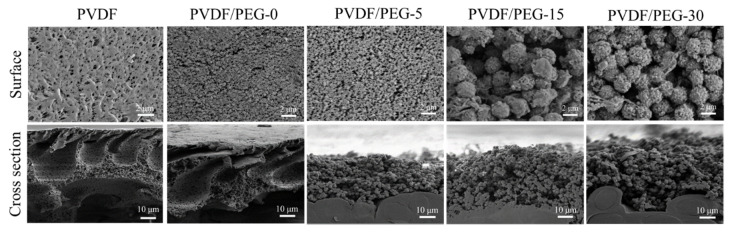
The FESEM images of the top surface and cross-sectional morphology of the resulting membranes at a magnification of 3000× and 1000×, respectively.

Figure 3 shows the morphological images of the developed membranes. The results suggest a strong effect of exposure time on the resulting PVDF membrane morphology. A typical asymmetric structure with a dense top layer was obtained when the membrane films were cast using the NIPS method (immediately immersed after casting), as shown by the (pristine) PVDF and PVDF/PEG-0 membranes. Based on the cross-sectional FESEM images, both membranes were asymmetric and had large macrovoids with a finger-like substructure near the top surface, suggesting that they underwent instantaneous demixing in which the polymer precipitates and a solid film was formed very rapidly after immersion in the nonsolvent bath [12]. The formation of such a structure can be ascribed by the thermodynamic and kinetic aspects of the liquid–liquid phase demixing process [25]. Precipitation takes place due to the low miscibility between the polymer (PVDF) and nonsolvent (coagulation bath/water). At the same time, the miscibility between solvent (DMAC) and nonsolvent (water) also causes the exchange/diffusional flow of solvent–nonsolvent at several points of the film’s top layer and sublayer [11]. This phenomenon leads to the formation of nuclei of a polymer-poor phase, which subsequently causes the formation of macrovoids. Some authors attribute that the initial point of macrovoid is due to the rupture of the thin top layer caused by the mechanical stress after immersion of polymer film [26]. 

It is worth noting that the macrovoids formation in PVDF/PEG-0 are smaller compared to the pristine PVDF membrane. It demonstrates that the macromolecular properties of PEG are responsible for the suppression of macrovoids formation. This is due to the presence of two polymers in the same solution that have to diffuse with respect to each other in order to phase separate and create a different time scale for the film formation [27]. Since the PEG possesses properties that are similar to nonsolvent, its presence enhances thermodynamic instability of the cast film and leads to instantaneous demixing [10,11]. The top surface morphology shown by FESEM images suggests that the PEG leads to the formation of a porous layer with a small solid and granular structure on the membrane surface, as shown by the PVDF/PEG-0 membrane. This is in line with studies reported by Kim et al. [10], where the membrane top layer becomes more porous with the increasing amount of PEG. 

For the membranes involving in the VIPS process, their morphology changes drastically from the typically asymmetric to microvoids-free, solid sphere and granular structure, which is also known as a symmetric nodular structure [7,20,28] as shown by PVDF/PEG-5, PVDF/PEG-15, and PVDF/PEG-30. The sudden change in morphologies suggests that the nonsolvent water vapor from the air has ample time to imbibe to the cast film. The formation of solid sphere morphologies is commonly found for VIPS-based membranes [29]. The slower uptake of the water vapor from air favors a polymer crystallization (solid-liquid demixing) process over the liquid-liquid demixing [25]. The process ensured the cast film in the crystallization region without being affected by liquid-liquid phase separation. The PVDF polymer crystals dissolve in the solvent to form a homogeneous polymer solution, while the present small amount of dissolution with a very small crystalline nucleus would serve as the initial nuclei during recrystallization process [30]. The entangled PVDF polymer nuclei chain crystals are favored by the liquid-solid separation and are further enhanced by the VIPS process, which results in the formation of the solid spherulitic nodular structure [31].

When the exposure time is increased from 5 to 30 min, the size of the sphere’s structure also increases. The polymer nuclei density is constant at constant temperature since the small crystalline entities (or nuclei) are stable over time [32]. Therefore, at shorter exposure time, the available time is limited for the growth of nuclei, resulting in smaller nodules structures; but in return, they are more interconnected and thus resulted in better mechanical properties. In addition, this study also shows that membrane thickness increases with the extension of exposure time to give values of 148.62 ± 2.31, 147.20 ± 4.12, 145.54 ± 0.2, and 139.80 ± 0.81 μm for PVDF/PEG-0, PVDF/PEG-5, PVDF/PEG-15, and PVDF/PEG-30, respectively.

#### 3.1.2. Membrane Pore Size and Distribution 

**Figure 4:**
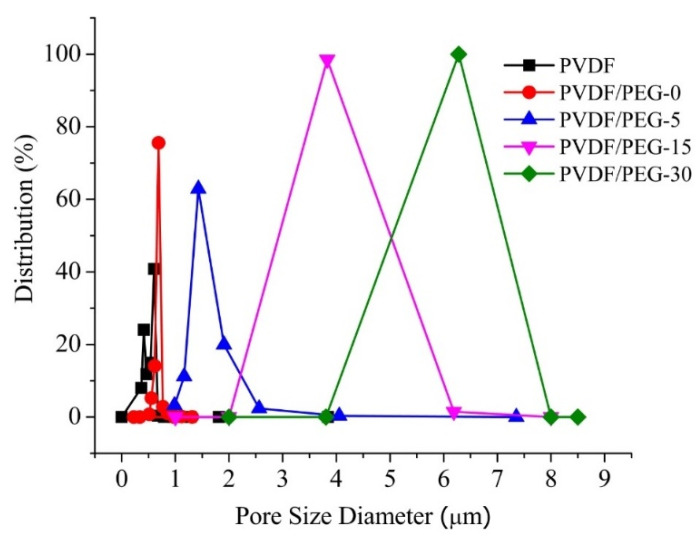
The pore size distribution of the resulting membranes.

Figure 4 depicts the pore size distribution of the developed membranes. It is found that the incorporation of PEG and LiCl slightly improves the membrane mean pore size from 0.58 ± 0.24 μm (pristine PVDF membrane) to 0.74 ± 0.02 μm (PVDF/PEG-0). However, applying VIPS method obviously increases the membrane pore size; and extending the exposure time before immersion would significantly enlarge the membrane pore size as shown by PVDF/PEG-5, PVDF/PEG-15 and PVDF/PEG-30 with their respective mean pore size of 0.74 ± 0.02, 1.85 ± 0.11, 6.06 ± 1.42, and 9.34 ± 1.57 μm. The increasing trend of the pore size can be justified as an effect of slow nonsolvent penetration process, which favors solid-liquid demixing. Nucleation dominates the polymer lean phases and contributes to the formation of a highly porous membrane. The pores formed as the voids between the spheres are thus higher when the matrix is formed by larger spherical sizes [33,34].

The slight difference in pore size of PVDF and PVDF/PEG-0 membranes can be explained by the role of PEG and LiCl as the additives in pronouncing the impact of thermodynamic rather than the kinetic. Their presence increases the thermodynamic miscibility of the solvent and nonsolvent, which enhances the rate of liquid–liquid demixing [35]. However, it also increases the viscosity of the casting solution, which slows down the demixing rate. Despite the instantaneous process, relatively slower demixing leads to the formation of a sponge-like structure in the membrane sublayer [36]. In general, the addition of hydrophilic additives to the casting solution has a dual effect on the membrane morphology: (i) facilitates the formation of macrovoids due to intensification of thermodynamic instability and (ii) suppress macrovoids formation due to increased viscosity of casting solution, as detailed by others [11,37]. 

The increase of membrane pore size with the extension of exposure time might also be due to the presence of PEG additive. More PEG chains move to the membrane/water interface, resulting in larger mean pore size, as reported elsewhere [7]. Besides, the high mutual affinity of the solvent and nonsolvent system allows the formation of a highly porous membrane as instantaneous demixing tends to occur [12]. 

#### 3.1.3. Surface Contact Angle

**Figure 5:**
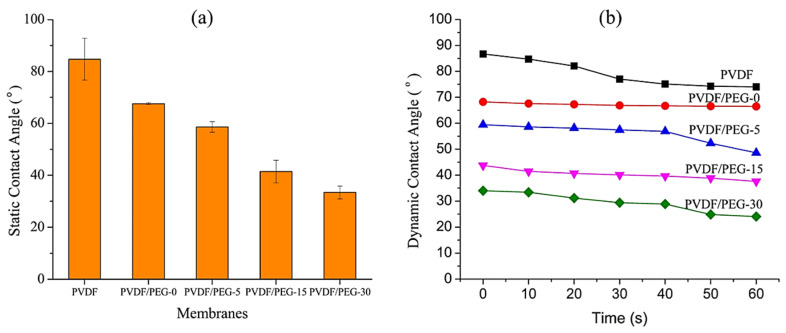
The static (**a**) and dynamic (**b**) contact angle of the resulting membrane.

The addition of PEG into the polymer solution clearly influences the membrane morphology by influencing the pore formation and structures as well as membrane hydrophilicity [38]. Contact angle data in Figure 5 demonstrates that the pristine PVDF membrane shows the most hydrophobic properties with a static contact angle of 84.72 ± 8.07, which is consistent with reported literature [39,40,41,42]. As expected, the membranes relatively become more hydrophilic when the exposure time was lengthened. It is demonstrated by the decreasing values of static contact angle for each membrane (Figure 5a) which are from 67.6 ± 0.37, 58.6 ± 2.02, 41.44 ± 4.33 to 33.41 ± 2.47 for PVDF/PEG-0, PVDF/PEG-5, PVDF/PEG-15, and PVDF/PEG-30, respectively. This finding is encouraging and supports the hypothesis developed in this study since membrane fouling propensity can be reduced with a hydrophilic surface. The hydrophilic surface enables the formation of a hydration layer between water molecules, and hydroxyl groups act as a physical barrier for foulant/membrane interaction [43]. 

The increasing trend of membrane surface hydrophilicity with respect to exposure time might be attributed to the higher amount of PEG residual resides within the membrane matrix, as proven by the chemical analysis results (in Section 3.1.4). Even though a fraction of PEG leaches out during phase inversion process, some remained in the membrane matrix, most likely due to partial formation of semi-solid structure on top of the film during the exposure time, as hypothesized in this study. The immobile structure pre-formed on top of the cast film during the exposure time acted as a barrier for PEG that hindered its leaching during the immersion. This phenomenon can be further explained due to the slow nonsolvent intake rate feature that makes the VIPS method prefer solid-liquid demixing, resulting in limited mobility of the polymer chains in the system as the phase separation takes place accordingly [7]. Once the mobility of the polymer chains decreases, the diffusion of the antifouling agent was limited by the fixed polymer chains in the polymer-rich domain, thus restricting the diffusion of PEG through the PVDF polymer matrix. 

#### 3.1.4. Fourier Transform Infrared (FTIR)

**Figure 6:**
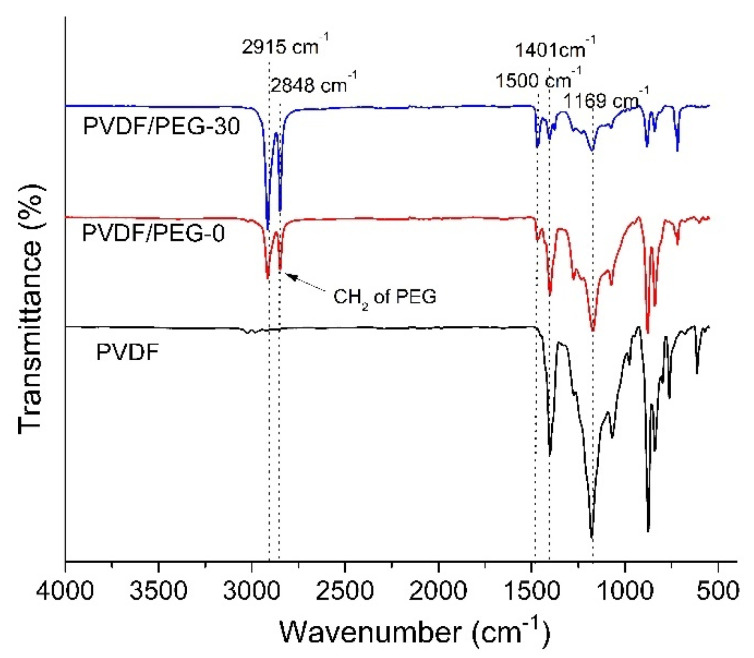
FTIR spectra of the selected membranes.

The FTIR spectra in Figure 6 signifies the positive effect of the VIPS process in enhancing membrane surface hydrophilicity. The appearance of peaks at 1401 and 1169 cm^−1^ in all tested membrane samples corresponds to CH_2_ stretching and C–F stretching vibrations from the PVDF chain, respectively. The pristine PVDF membrane has a stronger peak intensity at a wavenumber of 1169 cm^−1^ (C–F stretching bonds in PVDF polymer) compared to the modified membranes. The appearance of a new peak at the wavelength of 1500 cm^−1^ can be attributed to the CH bending of PEG residual, which typically occurs at 1467 cm^−1^ [44].

Knowing that FTIR results also can assist in quantifying the amount of residual PEG in the membrane matrix, it is interesting to find that new peaks were observed at 2915 and 2848 cm^−1^ upon addition of hydrophilic additive which corresponds to stretching vibration of aliphatic CH_2_ in the PEG. According to Marbelia et al. [45], hydrophilic additives such as PVP and PEG could leach out from the cast film to the nonsolvent during the phase inversion process. However, the trend suggests that the intensity of peaks displayed at these wavenumbers (2915 and 2848 cm^−1^) by both PVDF/PEG-0 and PVDF/PVDF-30 signify the presence of the PEG residual. Meanwhile, the peak intensity depicted by PVDF/PEG-30 is higher than the PVDF/PEG-0, which means that a longer time gap leads to a higher residual of PEG in the membrane matrix. It seems that the prolonged exposure time allows the formation of semi-solid form on the top layer of the cast film, which hinders the leaching of PEG from the polymer matrix. In the phase inversion process, small molecules PEG is easily washed out when immersion in nonsolvent due to the high diffusion rate. Due to its larger molecular structure of PEG (200 kDa) than the solvent (DMAC, molar mass of 87.12 g/mol), the diffusion rate of the PEG molecule from the polymer-rich phase to the polymer lean phase is much lower than that of the solvent. Therefore, their mobility to the surface of the film is restricted as such some PEG remained entrapped in the membrane matrix [46].

#### 3.1.5. Surface Chemical Composition

**Figure 7:**
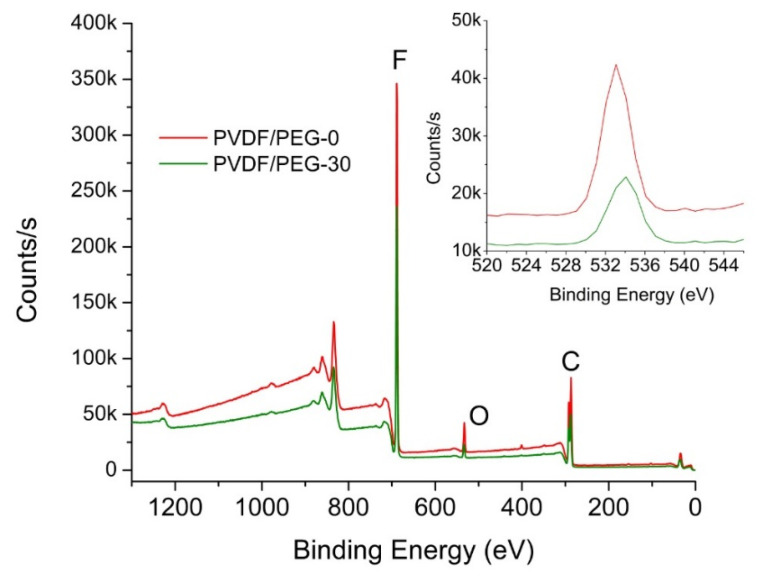
XPS wide scan spectra of PVDF/PEG-0 and PVDF/PEG-30 membranes (inset shows the corresponding intensity of selected element).

The distribution of elemental composition obtained using EDX mapping is summarized in Table 1. It shows that the oxygen originated from the hydroxyl group in the PEG increases at higher exposure time, suggesting its positive impact in the preservation of PEG. A longer exposure time allows the polymer chain to set up PVDF-rich segments that entrap the PEG that can no longer diffuse towards the polymeric system/nonsolvent interface and thus limits its leaching [7]. In contrast, the elemental composition obtained from XPS shows a slightly different result (Figure 7). It suggests that the oxygen composition of the PVDF/PEG-0 surface is 4.41%, which is slightly higher than PVDF/PEG-30 (3.34%). The minor variation might be because of XPS is a highly surface sensitive tool and thus unable to detect the PEG entrapped in the polymer segment (see spherulitic structure in Figure 3) across the thickness of the membranes of 100–200 μm and it is worsened when the polymer is in spheritic nodules with poor connectivity [7]. So, unless the membrane is very thin, it remains challenging to explain and characterize the surface segregation using XPS data.

#### 3.1.6. Clean Water Permeability

**Figure 8:**
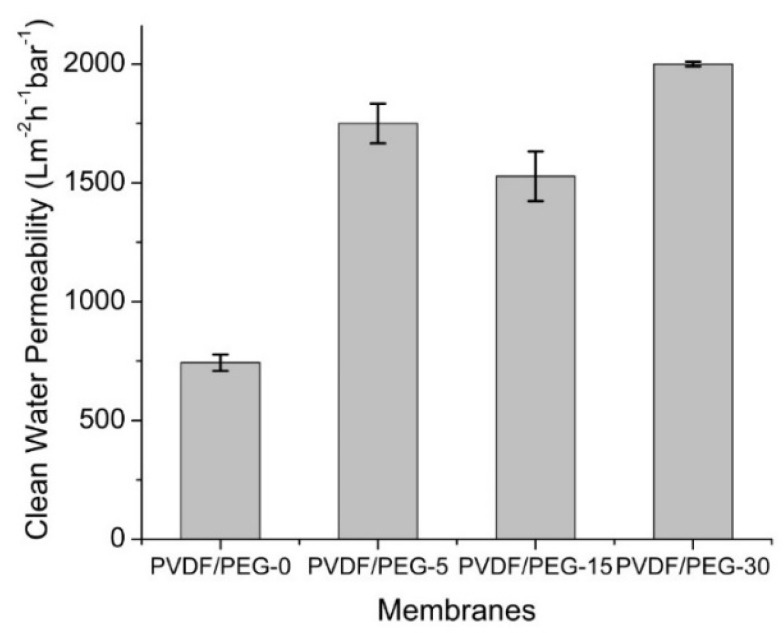
Clean water permeability (CWP) of the resulting membranes.

Figure 8 shows the CWPs of the developed membranes demonstrating the clear effect of the VIPS method and exposure time in lowering the intrinsic membrane resistance. The relatively low CWP obtained by the PVDF/PEG-0 membrane is due to its smaller mean pore size (Figure 4), which restricts the water transport across the membrane film. Membrane hydraulic performance is closely related to the membrane structure. The porous substructure with large macrovoids possessed by PVDF/PEG-0 membrane contributes to a larger degree of membrane compaction compared to the rest. According to Persson et al. [47], a membrane with macrovoids structure is highly affected by compaction than the one with a sponge-like structure. The compaction occurs due to the applied TMP during filtration that squeezes the porous structure changing, thereby the permeability and the selectivity of the membrane [48]. 

Besides, the higher content of PEG on the membrane surface facilitates the movement of water molecules across the membranes and generally improves the CWP. The higher CWP shown by the membranes developed using the VIPS method also might be related to more interconnected pores that would assist mass transfer. In addition, it is worth noting that the combined effect of membrane structures, including lower membrane thickness, larger pore size, and higher porosity would greatly enhance the membrane permeation. 

### 3.2. Effect of Exposure Time on Membrane Hydraulic Performance

#### 3.2.1. Permeance Recovery Analysis

**Figure 9:**
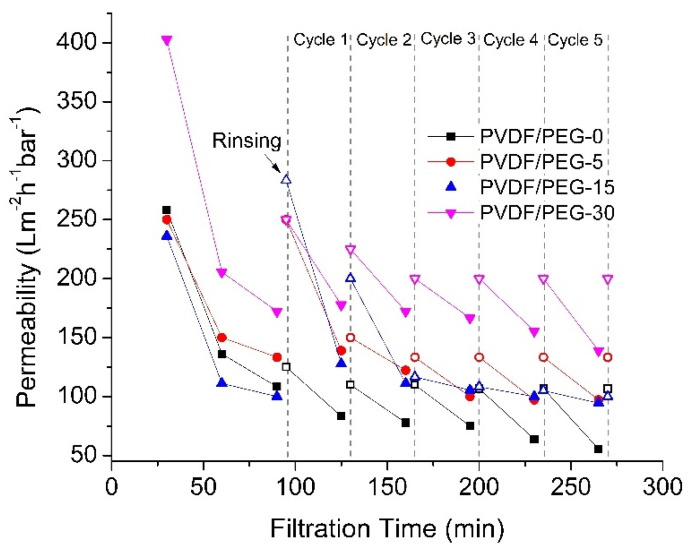
Permeability of the developed membranes treating PW with frequent water flushing.

Figure 9 shows that the permeabilities of all membranes decline over filtration time but with different extents. The first part of filtration was performed using PW as feed for 90 min before being flushed with distilled water for cleaning purposes. The steady-state permeabilities of PVDF/PEG-0, PVDF/PEG-5, PVDF/PEG-15, and PVDF/PEG-30 obtained from the first part of filtration are 122.22 ± 19.64, 141.67 ± 11.79, 105.56 ± 7.86, and 188.89 ± 23.57 L·m^−2^h^−1^bar^−1^, respectively. Before continuing with the next PW filtration, the CWP of the membrane was measured, to complete one filtration cycle. The depreciation of membrane permeability over time demonstrates that it is impossible to totally avoid membrane fouling; it can only be delayed with proper fouling control [49]. Generally, the permeation trend from this study suggests that membrane cleaning by water flushing able to reduce the decreasing rate of permeation and prove its efficiency to reduce the effect of membrane fouling.

Figure 9 demonstrates that the PVDF/PEG-30 shows the highest permeability for the first 90 min of filtration, and it is maintained until the end of the following filtration cycles. The high performance of the PVDF/PEG-30 can be attributed to the presence of PEG on the membrane surface that imposes surface hydrophilicity, which is beneficial for repelling oily foulant when treating the PW. The antifouling surface is then coupled with high porosity and the least thickness to offer a better permeability compared to other membranes. In the meantime, studies show that membrane with higher flux most probably suffers from more severe membrane fouling due to the greater convective force that brings about foulants to the membrane surface [50]. However, despite relatively high permeability shown by PVDF/PEG-30, it exhibited a high permeability recovery of 37% at the fifth filtration cycle that delays the effect of membrane fouling. 

In contrast, the fouling effect is more pronounced for PVDF/PEG-0 membrane. Compared to PVDF/PEG-30 membrane, PVDF/PEG-0 exhibits low initial permeability since it suffers the most from membrane compaction during earlier filtration that might alter the initial membrane structure and diminish the pathway for mass transfer. The least hydrophilicity properties of the membrane also may attract the hydrophobic oil constituents in the PW feed and cause foulant accumulation on the membrane surface. The layer of foulants block the available pores for mass transfer and consequently diminish its permeability performance. Therefore, the membrane obtained via the NIPS process in which PEG is mostly leached has lower hydrophilicity property and would result in lower permeability, as also consistently reported in the literature [10,38,51]. Fortunately, the water flushing introduced at each filtration cycle was able to remove the deposited particles and reduced the fouling effect.

#### 3.2.2. Rejection Performance

**Figure 10:**
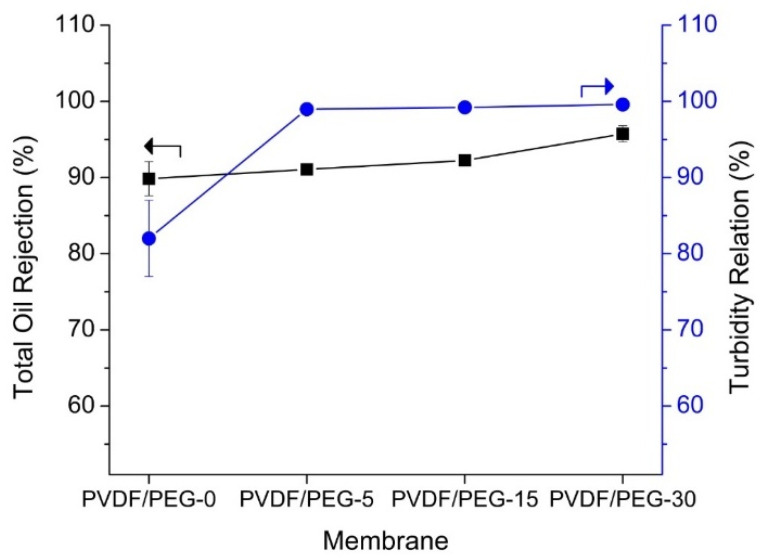
The quality of permeate in terms of oil rejection and turbidity obtained after 1.5 h of PW filtration.

Figure 10 shows all membranes achieve high oil rejection of >90%. Membranes prepared using VIPS method with longer exposure time before immersion demonstrate better oil rejection. The separation of the emulsified oil could be highly affected by the presence of PEG despite large membrane pore size. Similar findings were also reported by Zhang et al. [52] that attributed good rejection due to a spherical structure that promotes efficient and fast separation of oil-rich emulsions. The permeability has also increased by a factor of four with a separation efficiency of 99.99% for VIPS-based membrane when separating water/toluene emulsion according to a study performed by Venault et al. [53]. This further justifies our results that prolonged exposure time leads to higher oil rejection efficiency together with high permeability. Preparation of membrane with >30 min time gas was not possible due to very weak physical properties that could not withstand the low pressure applied in the study. 

#### 3.2.3. Fouling Resistance Analysis

**Figure 11:**
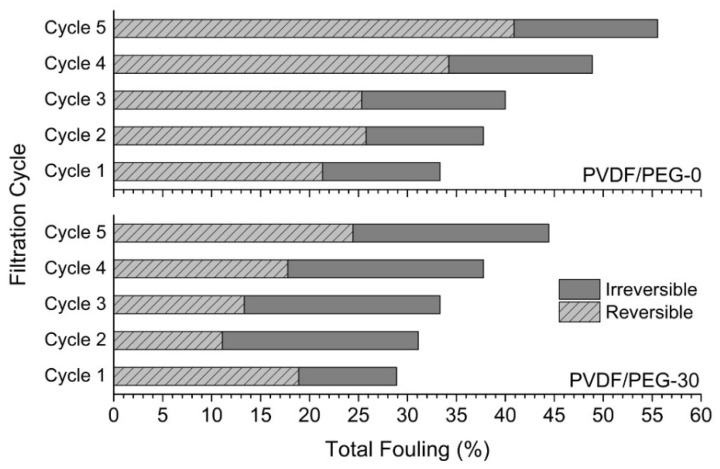
Fouling resistance analysis of modified membranes developed using NIPS (PVDF/PEG-0, up) and VIPS (PVDF/PEG-30, bottom) techniques.

Figure 11 shows the effect of VIPS method on the membrane antifouling properties, which was evaluated based on their reversible and irreversible fouling. Only the results for PVDF/PEG-0 and PVDF/PEG-30 are included in the analysis since both membranes show the most obvious findings, as depicted in the previous sections. In general, total fouling for both membranes increase with the increase of the filtration cycle. However, the total fouling exhibited by PVDF/PEG-30 at each cycle is relatively lower than PVDF/PEG-0 membrane, demonstrating that the former has better antifouling properties with a stronger ability to restore CWP of the fouled membrane. This observation is also as expected as a membrane with low PVDF/PEG-0 likely causes more fouling due to the rapid compaction of the fouling layer and pore-clogging [54]. Membrane fouling commonly can be divided into reversible and irreversible types of fouling, which the former can be eliminated by physical cleaning such as water flushing while the latter require chemical cleaning. 

For PVDF/PEG-30, it is interesting to find that the membrane only suffers from a relatively high degree of irreversible fouling starting from the second cycle. We believe that this condition is related to the high PEG content that resides in the membrane matrix, causes PEG swelling that compress the membrane pores and diminish the permeance. Besides, at the second filtration cycle, the membrane suffers from about 20% of irreversible fouling, and it is maintained until the last cycle, which supports the argument. At the same time, it should be noted that high irreversible fouling may cause a greater degree of performance loss. The results obtained also highlight that the presence of hydrophilic functional groups in the composite membrane could increase the intermolecular hydrogen bonding of membrane surface with the water molecules, and at the same time, weaken the hydrophobic interaction between oil droplets and the membrane surface effectively. This is beneficial for preventing foulant build-up. Similarly, previous studies suggested that the increase in surface hydrophilicity of the membrane would result in improved flux recovery with a lower fouling rate [2,55,56,57]. However, since the degree of fouling is evaluated based on the loss of permeability, with our limited setup, it is difficult to prove that the permeance deterioration of PVDF/PEG-30 is due to permanent attachment of foulants or alteration of membrane structure caused by the PEG swelling.

The total fouling experienced by PVDF/PEG-0 is dominated by reversible fouling, which develops due to the accumulation of foulants whose particle sizes are larger than the membrane pore size. Thus, the relatively small mean pore size possessed by PVDF/PEG-0 membrane elevates the degree of reversible fouling. Referring to Figure 11, PVDF/PEG-0 membrane shows about 12% of irreversible fouling at the second filtration cycle, and it slightly increases to 15% in the third cycle and maintains until the last cycle. A study by Tsuyura et al. [54] reported that contact angle and CWP influence the degree of physically irreversible fouling, suggesting that the irreversible fouling might correspond to membrane’s properties of relatively high surface contact angle (more hydrophobic) and low CWP. Le-Clech et al. [58] proposed that membrane with hydrophobic properties are expected to suffer from fouling more severe than hydrophilic membrane due to the hydrophobic interactions between the foulants and membrane material. The degree of fouling for the developed membrane further be reduced when coupled with hydrodynamic based fouling control methods, such as via membrane surface patterning [40,59], tilting panel [60], finned spacer [61,62], or combination thereof [63].

## 4. Conclusions

This study proves that the extension of exposure time using the VIPS method could improve the retention of PEG additives in the polymer matrix and minimize the leaching of PEG to the coagulation bath during immersion. The prolonged exposure time changes the membrane morphology and structure from finger-like macrovoids to spherical nodules structure. It also improves membrane surface hydrophilicity, enlarges mean pore size, increases clean water permeability, which enhances membrane hydraulic performance, and its antifouling properties. Such an impact on the membrane properties offers an advantage in the hydraulic performance for the treatment of PW. Extending the exposure time from 0 to 30 min elevates the steady-state permeability from 122 to 189 L·m^−2^h^−1^bar^−1^ while maintaining the quality of permeate with oil rejection of >90%. At the same time, exposing the cast film for 30 min before immersion improves membrane antifouling properties, and it is proven by the decline of total fouling at each filtration cycle compared to the NIPS-based membrane. For instance, its total fouling at the fifth filtration cycle is reduced by about 20% compared to the NIPS-based membrane, indicating that the membrane possesses greater fouling resistance. 

## Figures and Tables

**Table 1:** Chemical composition distribution of the PVDF membrane and modified membranes by EDX.

Membrane	Composition (at %)		
	C	F	O
PVDF/PEG-0	50.79	46.98	2.22
PVDF/PEG-5	51.37	45.38	3.25
PVDF/PEG-15	52.31	43.72	3.97
PVDF/PEG-30	57.78	37.25	4.97
